# Preclinical Studies and Translational Applications of Intracerebral Hemorrhage

**DOI:** 10.1155/2017/5135429

**Published:** 2017-06-18

**Authors:** Felix Siaw-Debrah, Mark Nyanzu, Haoqi Ni, Xiao Lin, Zhu Xu, Linhui Ruan, Qichuan Zhuge, Lijie Huang

**Affiliations:** ^1^Department of Neurosurgery, The First Affiliated Hospital of Wenzhou Medical University, Wenzhou 325000, China; ^2^Zhejiang Provincial Key Laboratory of Aging and Neurological Disorder Research, First Affiliated Hospital, Wenzhou Medical University, Wenzhou 325000, China

## Abstract

Intracerebral hemorrhage (ICH) which refers to bleeding in the brain is a very deleterious condition with high mortality and disability rate. Surgery or conservative therapy remains the treatment option. Various studies have divided the disease process of ICH into primary and secondary injury, for which knowledge into these processes has yielded many preclinical and clinical treatment options. The aim of this review is to highlight some of the new experimental drugs as well as other treatment options like stem cell therapy, rehabilitation, and nanomedicine and mention some translational clinical applications that have been done with these treatment options.

## 1. Introduction

Intracerebral hemorrhage (ICH) is a devastating disease and the second leading cause of stroke [1]. It accounts for about 25 per 100,000 cases annually and results in high mortality rate [2]. ICH episode results in primary injury to the brain which initiates other devastating cascades leading to further damage. Treatment options still remain to be either surgical intervention or conservative therapy [3]. Primary injury occurs from direct injury by mass effect of the hematoma or by neurovascular disruption. Treatment during primary injury is still debatable between hematoma evacuation and conservative treatment, since at this point attempt to evacuate hematoma might lead to further brain damage [4]. Hematoma expansion further aggravates primary injury within first 24 hours of icterus. Secondary damage to the brain occurs due to series of events that are initiated by primary injury and its metabolites. These include insult from blood cell lysis (iron, heme, etc.), thrombin cascade activation, and inflammation [5]. Thrombin is implicated in the initiation of cerebral injury after hematoma [6] and influences the degree of edema formation after ICH. Other studies have also identified thrombin to initiate blood-brain barrier (BBB) disruption [7], neuroinflammation, or neuroprotection at different quantities [8]. Different inflammatory cells and their mediators are involved in secondary damage. For example, mast cells and lymphocytes inhibition were seen to improve survival rate of experimental animals [9]. Inflammatory mediators like cytokines, matrix metallopeptidases (MMPs), and adhesion molecules have also been highlighted as causes of secondary brain injury [10–12]. Iron overload from lyses of red blood cells is another known factor of secondary injury, since it activates the ROS [13], leading to protein and DNA damage [14]. Disruption of the BBB allows proteins of complement system to easily cross into the brain. Crossing of activated complement cascade into the brain initiates the membrane attack complex (MAC) which further destroys the BBB allowing influx of fluid into the brain causing brain edema [15]. Perihematomal edema leads to mass effect and further aggravates secondary injury. Many clinical trials are therefore ongoing with some under completion, into different treatment options ideal for an ICH patient. The essence of this review is to highlight some of the current preclinical studies into various treatment options for ICH, further stating some translational applications and clinical trials.

### 1.1. ICH Disease Process

#### 1.1.1. Hematoma Enlargement

Enlargement of hematoma volume is seen to occur in about 30% of all patients [25], with about 72.9% of them having hematoma increase within the first 3 hours after incidence [26]. Hematoma may sometimes extend into the ventricles causing IVH (intraventricular hemorrhage) within first 24 hours after onset, in about 45% of all cases. A correlational study of IVH volume on prognosis predicted that a 2 ml increase in IVH within 24 hours indicated poor prognosis [27]. Other studies on the causes of hematoma expansion identified the shape of initial hematoma and the function of the liver to directly influence rebleeding. For example, Fujii et al. noted that patients with irregular shaped hematoma experience some degree of hematoma expansion compared to those with uniform shaped hematoma. They further noted that the same was true for patients with decreased fibrinogen and platelet count, as well as in patients with impair alpha 2-antiplasmin activity [28].

#### 1.1.2. Brain Edema

Edema formation around hematoma is commonly seen within hours to days after ICH. Edema formation can be divided into 3 phases. The first phase is characterized by difference in hydrostatic pressure between retracted clot and surrounding brain tissue. This phase begins several hours after ICH and is then followed by activation of coagulation pathway and thrombin release. This is mark of the second phase which last about 2 days after ICH. During the third phase (after 3 days) there is vast release of hemoglobin from hemolysis of red blood cells. Activation of the complement system is therefore a major contributing factor to edema formation during the second and third phase [29]. The process of edema formation can therefore be summarized into the following events: that is, mass effect, clot retraction, thrombin formation, and hemolysis of RBC, hemoglobin release, complement system activation, and breakdown of BBB. Hoff and Xi therefore made an important accession that early evacuation of hematoma could interrupt the process of edema formation [30].

#### 1.1.3. Neural Cell Death

Another devastating effect of ICH is neural cell death. During an ICH, reactive oxygen species (ROS) are released. These compounds initiate mitochondria-dependent or independent cell death pathways via oxidative stress [31]. Other studies have also identified that glutamate can affect the cell death. Glutamate production increases in brain parenchymal after an ICH episode triggering release of inflammatory cells and byproducts of erythrocyte breakdown. These products have been seen to be major free radical activators which in turn activate other cascades leading to cell death [32].

#### 1.1.4. Inflammation

The presence of hematoma in brain parenchyma triggers brain's resident microglia. Activated microglia further recruits other leukocytes causing excessive release of inflammatory mediators [33]. Pozzilli et al. demonstrated that inflammation following ICH involves both resident and migration of circulatory cells to the brain [34]. Wu et al. also studied the effect of inflammation on edema formation reiterating the expression of macrophage inflammatory protein-2 (MIP-2) to contribute largely to edema formation. They added that MIP-2 expression began 2 hours and peaks 2 days after hemorrhage. Furthermore, they found that MIP-2 mediated edema formation was mediated by NF-kappaB activation [35]. Other studies found that a WBC count of 10,000/mL^3^ could lead to early neurological deterioration within the first 3 days of ICH [33]. Inflammation therefore plays a major role in disease process of ICH.

#### 1.1.5. Recovery

After an ICH episode, some patients experience some extent of functional recovery although most patients live with some form of permanent disability. Many experiments have identified neurogenesis to account for the mechanism of recovery; Yang et al. found that thrombin formation can stimulate some amount of neurogenesis leading to some extent of functional recovery [36]. Similarly functional recovery is attributed to resolution of hematoma's mass effect, edema, and neuroplasticity of surrounding resident neurons [37].

## 2. Preclinical Studies of ICH: Models and Drugs

### 2.1. ICH Modeling

The constant tussle surrounding the treatment of ICH has called for many researches that seek to uncover the mystery behind the disease process. An ICH episode requires prompt attention and urgent intervention as such the study of ICH in the clinical setting is somewhat limited in patients. Studies on ICH could therefore be dependent on advanced imaging and other pathological studies. It is therefore prudent to model ICH in the attempt to mimic what happens in human patients. Till date there are three main techniques that are widely used in the study of ICH: autologous blood injection, collagenase blood injection, and microballoon injection (Table 1).

The collagenase injection model is the commonest model in recent times. This model is created by injecting about 0.075–0.4 U of collagenase into the basal ganglia of striatum of the animal allowing the collagenase to dissolve and rupture small vessels of the brain. It is therefore useful in simulating deep brain and penetrating vessel ruptured hemorrhages [38–40]. The collagenase injection model has the possibility of inducing cellular toxicity [38]. Therefore, more damage is done to basal ganglia putting in question its ability to correctly mimic the human ICH [41, 42]. The autologous blood injection model is another commonly used model. This model injects about 50–100 *μ*L of blood into the striatum or cortex of the rat brain. This was the earliest type of model used to replicate lobar hemorrhage and also to study the mechanism of brain injury in perihematomal region [43, 44]. This model is unsuitable for studying hematoma enlargement due to consistency of hematoma volume [40]. Another demerit of this method is the tendency of reflux of hematoma along needle trail. Another important setback is that excessive brain damage will occur during a speedy induction of blood [40, 45]. Mass effect was simulated and studied by Sinar et al., who inflated a microballoon into the rat's brain. This model was used to find out the effect of hematoma evacuation on the brain [46]. The microballoon model can only simulate mass effect without putting into consideration the pathological effect of hematoma like edema. There is also minimal damage caused using this model making it impossible to truly mimic what happens in humans [47].

Animal models can basically be divided into the small and large animal model. With regard to advantages, the small animal model is cheaper due to cheaper cost of raising animals and is also easy to model. Small animals have short gestational period making experimental time shorter. It is therefore convenient for immunohistochemical and biochemical studies. The large animal model however presents the advantage of being close in anatomical structures and genetic composition to humans. They have large gyrus and well differentiated white matter making replication of hematoma in large animal mimic humans more accurately. The replication of a successful small animal model in the large animal model will therefore potentiate the studies chances of being translated into the clinic.

### 2.2. Preclinical Drugs for Treating ICH

**Figure 1:**
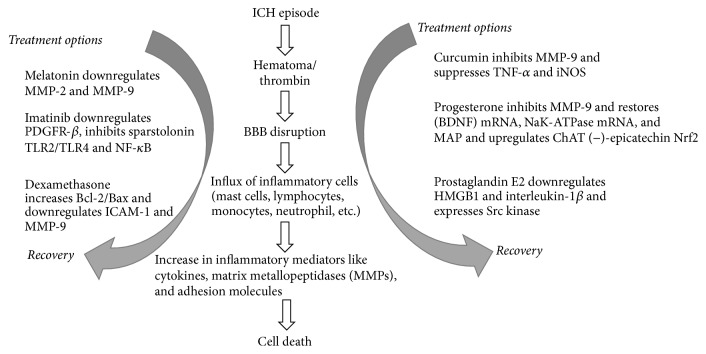
Preclinical drugs and their targets in ICH disease process.

There have been many studies done on different drugs which have been proven in small animal models to be efficient in treating ICH (Figure 1). There is therefore the need to experiment more of these drugs in large animals to enable translation into clinical trials.

#### 2.2.1. Treating Perihematomal Edema

*Neuroinflammation and Oxidative Stress Drugs*

*Curcumin*. Curcumin is a yellow pigmented polyphenol derivative of curcuma longa. It is widely used as coloring agent but has also been found to be useful in the treatment of various diseases [48]. Due to its antioxidant, anti-inflammatory, antiviral, antibacterial, antifungal, and anticancer properties, curcumin has been studied as potential therapy for many infectious and cancerous diseases [49]. Curcumin is hypothesized to be useful in attenuating hematoma size and reducing inflammation after hemorrhage. According to Fu and Kurzrock, curcumin influences biological processes either through direct impact on target protein or through epigenetic modulation, that is, the modulation of genetic environment without affecting gene structure [50]. Curcumin also possesses additional characteristics of being able to cross the BBB as well as having lower toxicity even at high dosages making it suitable for treatment of cerebral diseases. The optimum dose for maximum effect of curcumin was discovered by King et al. to be 150 mg/kg. King et al. in the same experiment found the therapeutic window of curcumin to be within 1–72 hours after injury with maximum efficacy within first 3 hrs [51]. Another study on curcumin's effect on ICH demonstrated its potential in attenuating edema via protection of BBB integrity after an ICH. The integrity of BBB is compromised during cerebral hemorrhage allowing for excessive influx of fluid and protein causing massive edema. Curcumin is able to inhibit the expression of MMP-9 and ionized calcium-binding adapter-1 (iba-1) positive microglia and strengthen the integrity of BBB by enhancing occludins and zonula occludens (ZO-1) expression [52, 53]. This effect will therefore lead to the inhibition of perihematomal edema and prevent further damage to the brain. Other studies on curcumin reported its function in reducing excessive inflammation and injury to the brain [54]. Curcumin treatment has also been seen to result in improvement in learning and memory functions after curcumin administration. This is due to curcumin's ability to suppress release of TNF-*α* and iNOS in the hippocampus consequently improving cognitive function [55]. Curcumin could therefore be used to reduce ICH related inflammation and edema and improve neurologic function. There is the need for further studies into its usage as a treatment option after ICH.

*Progesterone*. Progesterone is a steroid hormone with a well documented role in menstruation. An abnormality with this hormone therefore leads to menstrual abnormalities in female. Progesterone is produced by the ovaries but also believed to be a product of neurosteroids pregnenolone and dehydroepiandrosterone [56]. In animal studies, ICH deleterious effect on female animals was discovered to be somewhat masked due to the presence of progesterone. This raised the belief that progesterone could be a useful therapeutic option for ICH. Progesterone has been found to exert neuroprotective effect by attenuating inflammation and improving neurologic outcome. A study by Jiang et al., to determine the effect of progesterone on neurologic outcome, deduced from histopathologic results that progesterone reduces brain edema, decreases size of lesion, and attenuates neuroinflammatory activities. Furthermore, progesterone suppressed activity of MMP-9, carboxylation, and nitroxylation of proteins and suppressed other molecular inflammatory responses. The same study also discovered reduction in scaring tissue and brain tissue loss after progesterone application [57]. Similarly, the effect of progesterone on ICH was studied by Lei et al., who observed decreased edema formation, attenuation of neuroinflammation, and glial scar tissue formation after progesterone use. They also observed that these effects were heightened in male and aged female rats but were less obvious in young female rats [58]. Progesterone also restores the normal functioning of brain-derived neurotrophic factor (BDNF) mRNA, Na-K-ATPase mRNA, microtubule-associated protein (MAP) 2, and choline acetyltransferase (ChAT) [59]. Progesterone therapy could therefore serve as treatment option for post-ICH edema and inflammation. Additional studies are therefore warranted.

*(−)-Epicatechin*. (−)-Epicatechin (EC) is a natural flavonoid molecule found in high concentrations in green tea and cocoa. Consumption of EC rich products has been identified to reduce blood pressure and the incidence of cardiopulmonary diseases [60]. EC has also been known for its use in the treatment of diabetes and other liver and heart diseases. EC can easily cross the BBB and is also capable of attenuating oxidative/reductive stress through NF-E2-related factor (Nrf) 2 pathway [61]. Nrf2 pathway has been regarded as a protective agent for many organs in the body [62]. It is able to activate tissue protective factors and antioxidant genes which alleviate tissue damage [63–65]. EC in an experiment by Lan et al. using a wild-type and Nrf2 knocked-out mice indicated an upregulation of nuclear accumulation and superoxide dismutase 1 (SOD1) in wild-type mice but no change in SOD1 for Nrf2 knockout (KO) mice. In addition, (−)-epicatechin treatment decreased HO1 expression in Nrf2 KO mice but with no change in wild-type. They therefore concluded that (−)-epicatechin treatment prevents brain damage via Nrf2 upregulation and activator protein-1 (AP-1) inhibition with AP-1 inhibition independent of Nrf2 pathway [61]. (−)-Epicatechin will therefore provide another treatment option in attenuating inflammation after ICH.

*Prostaglandin E2*. Prostaglandins are arachidonic acid derivatives. They are synthesized through the cyclooxygenase pathway and regulates the process of inflammation, immune response, bone resorption and red blood cell production [66]. Prostaglandin mediated inflammatory response has been implicated in the exacerbation of ICH. Zhao et al. identified prostaglandin E2 receptor EP1 to be involved in inflammatory response, with its inhibition yielding favorable functional outcomes in animal models [67]. Another interesting finding by Liu and Sharp suggested that Src kinase expression served as a double edged sword which when activated in the acute phase leads to the disruption of the BBB and subsequent increase in edema formation, while in the chronic phase it mediates repair of the BBB reducing edema formation [68]. Other experimental findings identified acute blockade of Src kinase by an antagonist, prevented formation of brain edema, and at the same time prevented the repair of BBB during continual blockage for 2–6 days. This is because continual blockade of Src kinase leads to the downregulation of brain microvascular endothelial cell (BMVECs) and perivascular astrocytes necessary for BBB repairs [7, 69, 70]. Misoprostol is an analog of prostaglandin E2 which is reported to attenuate cerebral injury within 24 hours following insult [71]. Misoprostol in an experiment was seen to decrease brain edema and neuroinflammation and improve functional outcome. This was achieved via the downregulation of HMGB1, interleukin-1*β*, and Src kinase expression. Furthermore, misoprostol alleviates inflammatory cascades and oxidative stress related brain injuries [72]. Prostaglandin's anti-inflammatory property is a needed quality in the fight against ICH; further studies are therefore a necessity.

*Melatonin*. Indoleamine melatonin is a hormone secreted by the pineal gland responsible for regulating sleep awake pattern and neuroendocrine processes [73]. Melatonin function is related to its ability to activate MT1 and MT2 receptors. Melatonin has been shown to reduce postischemic reperfusion related injuries like hemorrhages via downregulation of MMP-9 and MMP-2. Hemorrhages associated with postischemia could therefore be attenuated using melatonin treatment [74]. Lekic et al. also demonstrated that lower doses of melatonin at 1 and 24 hours for 3 days will lead to improvement of memory and striatal function after 8 weeks via neuroprotection and reduction of oxidative stress [75]. Although melatonin has effect on oxidative stress, its effect on brain edema formation is still under question. For instance, an experiment studying the antioxidative properties of melatonin revealed that melatonin had no effect on edema formation [76]. Melatonin has also been studied to improve electrical response to signal around hematoma region. Ueda et al. found the protection of oligodendrocytes and astrocytes via oxidative stress attenuation around hematoma region to be responsible for this behavior [77]. Melatonin therefore promises another alternative in the treatment of ICH and requires further studies.

*Imatinib*. Post-ICH inflammation is a common phenomenon after an ictus. Macrophages express platelet-derived growth factor receptor-*β* (PDGFR-*β*) and its agonist platelet-derived growth factor receptor-D (PDGFR-D) during ICH. This triggers an inflammatory cascade leading to further recruitment of other macrophages which causes neuroinflammation [78]. Inhibition of PDGFR-*α* has therefore been thought to be a therapeutic target for preventing inflammatory injury [43]. Imatinib a tyrosine-kinase inhibitor has been used over the years for the treatment of tumors and other bone malignancies. In neurological studies, imatinib has been studied to attenuate cerebral injury through inhibition of PDGFR-*α* [79]. In a research to validate the effect of imatinib on vasospasm, imatinib was seen to prevent vasospasm 24–72 hours via the downregulation of PDGFR-*β*, mitogen-activated protein kinase, TNC, and the deactivation of PDGFR [80]. Treatment of ICH with imatinib via inhibiting PDGFR promises to be an important therapy option that needs to be delved into.

*Sparstolonin B (Toll-Like Receptor Inhibitor 4 (TLR4))*. Toll-like receptors are pattern-recognizing receptors that recognize exogenous and endogenous molecular patterns initiating either adaptive or innate immunity [81, 82]. TLR4 has been implicated in inflammatory response after ICH. An upregulation of TLR4 has been reported in some studies with results indicating deterioration in condition after ictus in animal model [83]. Sparstolonin B (SsnB) is a* Sparganium stoloniferum* derivative which is studied to selectively inhibit TLR2/TLR4 making it useful in the treatment of many inflammatory diseases [84]. Zhong et al. demonstrated that Sparstolonin B could serve as a suitable treatment for ICH. Their results indicated inhibition of TLR2/TLR4 heterodimer formation thereby inhibiting secondary injury after ICH. Activity of SsnB was however dosage dependent with higher dosage (50 *μ*mol/L) giving the highest efficacy. Furthermore, the same experiment indicated that SsnB inhibited NF-*κ*B activity via TLR2/TLR4 heterodimer formation giving indication of SsnB's capacity in attenuating inflammation [85]. SsnB could therefore be used as a neuroprotective agent to alleviate ICH related inflammation. Additional studies should be conducted to understand SsnB therapy in detail.

*Dexamethasone*. Dexamethasone treatment is used in the clinic for both spinal cord injuries and tumor to reduce edema formation [86]. Its application in the treatment of ICH has also received massive research. For instance, the effect of dexamethasone was studied in a rat model immediately after ICH and 3 days after ICH induction. The experimental results revealed drastic reduction in edema formation through increase in the Bcl-2/Bax ratio and downregulation of cleaved caspase-3 which are known indicators of inflammation in ICH [87]. Other studies have revealed the effect of DEX in treating edema via the regulation of intercellular adhesion molecule-1 (ICAM-1) and matrix metalloproteinase-9 (MMP-9) expression [88]. Other studies on the efficacy of different doses of DEX on edema proved that, even at smaller doses, dexamethasone was still able to attenuate edema formation. For instance, experimental studies to find out the effect of different doses of dexamethasone on edema resolution concluded that lower doses of DEX (1 mg/kg) had beneficial effect on brain edema [89]. DEX could therefore be used as a treatment drug for attenuating inflammation and edema after an ICH episode. Further studies on the mechanism of action, including side effects, are needed.

#### 2.2.2. Treating Hematoma Growth

*Plasma Kallikrein Inhibitor (Aprotinin)*. Plasma kallikrein-kinin system (KKS) is made up of proteins factor XII (FXII), prekallikrein, and kininogen. The function of KKS includes regulating blood pressure, angiogenesis, inflammation, and cell proliferation and death. KKS is also strongly associated with coagulation, fibrinolysis, and vascular permeability. Experimental studies of KKS showed increased permeability of BBB accompanied by cerebral edema [90]. KKS activation increases vascular permeability and leads to blood extravasation from capillary causing hematoma enlargement [91]. Plasma kallikrein (PK) has been studied to increase hematoma volume in hyperglycemic rat model. Activation of PK affects collagen induced aggregation of platelets but has no effect on thrombin induced pathway [92]. A different study on the effect of prekallikrein on hematoma expansion after tPA treatment further stressed on the effect of plasma kallikrein (PK) on rebleeding. Thus the study concluded that inhibition of PK could serve as a therapeutic target to control rebleeding associated with tPA usage [93]. Aprotinin is a Kunitz-type protein and a known inhibitor of trypsin, plasmin, and both tissue and plasma kallikrein. Effect of aprotinin and its recombinant variant form on hematoma enlargement was done in rat subdural hematoma model. Experimental results showed that decrease in plasma kallikrein reduced vascular permeability and blood extravasations thereby reducing perihematomal edema and hematoma enlargement [94]. The use of aprotinin to reduce rebleeding is a possible therapy for ICH. Till date not many experiments have been done to explore the PK inhibitor as a treatment target of ICH; further studies are therefore needed.

### 2.3. Other Preclinical Therapies

#### 2.3.1. Neuroprotection and Functional Recovery

*Nanomedicine*. Nanomedicine refers to the use of nanoparticles in the treatment of disease. Several studies on the use of nanotechnology in the treatment of neurological disease have been conducted. The primary advantage of nanoparticles as carriers is to prevents the peripheral toxicity. The function of mitochondria is determined by the microviscosity of its membrane, which is affected by ischemic injury. In an experiment nanocapsulated-quercetin was seen to give protective effect to the mitochondria wall. The effect of nanocapsulated-quercetin on inducible nitric oxide synthase (iNOS) was further studied by Ghosh et al. who indicated that oral nanocapsulated-quercetin was able to downregulate iNOS which is expressed in ischemic cells compared to free quercetin administration. This further proved that nanocapsulated agents presented with efficacy and specificity as compared to their respective free agents [95]. In another experiment to demonstrate the effect of free quercetin as against nanoloaded quercetin's effect after ICH, nanocapsulated-quercetin proved better efficacy in reducing hematoma size, preserving glutathione S-transferase (GST) activity, and exhibiting total antioxidant properties [96]. Rapid functional recovery was therefore seen in nanocapsulated alarming researchers on the probability of using nanoparticles as treatment option for ICH. The use of self-assembling nanoparticles (SANP) in the treatment of ICH was also studied. This study identified SANP to reduce cavity formed after hematoma resolution and also improve functional recovery. SANP was seen to be compatible with local brain tissues and was therefore able to be transplanted into hematoma region reducing brain cavity [97]. The neuroprotective effect of poly(lactic-co-glycolic) acid (PLGA) nanoparticles loaded with recombinant human erythropoietin (rhEPO) was studied in hemorrhagic stroke model. The results indicated some level of functional recovery with decrease in brain damage [98]. Similar effect was seen in another experiment by Balaban'ian et al. who found improvement in rat model after rhEPO-loaded PLGA [99]. Nanoparticles application in the treatment of ICH presents a promising treatment option that should be looked at.

#### 2.3.2. Treating Neural Death and Neuroinflammation

*Enhancing Endogenous Neurogenesis*. The subventricular zone produces most of the neural cells that migrate along olfactory bulb. The extent of migration is however dependant on the presence of focal injury. Various experiments have shown that during injury SVZ cells migrate to the injured site and differentiate into glial cells [100, 101]. It is on this basis that many researchers have focused on how to initiate neurogenesis and increase migration of SVZ cells to treat neurogenic diseases. Many different pathways have therefore been researched into with the aim of initiating neurogenesis for which activation of macrophage/microglia seems to be one. In vitro study by Walton et al. found microglia to play significant role in neurogenesis. Their study indicated that activated microglia produces necessary factors that induce neurogenesis but do not induce neural cell proliferation themselves [102]. Another study about the role of microglia and T-lymphocytes in inducing neurogenesis discovered that, in an immune deficient model, neurogenesis was impaired even in a conducive microenvironment, further stressing on the importance of inflammation in neurogenesis [103]. Similarly, activated microglia turns to inhibit the expression of TGF-beta 1 an endothelial proliferation inhibitor in normal brain and upregulate TNF which is an angiogenic factor in injured brain [104]. This is due to the release of proangiogenesis factors VEGF and IL-8 by activated microglia. Angiogenesis could also be induced by neurotrophic, growth factors, anti-inflammatory drugs, hormones, and noncoding RNA. For instance, intrastriatal injection of glial cell line-derived neurotrophic factor (GDNF) has been seen to induce neurogenesis after ischemia [105]. Similarly, IGF-1 and GDNF have been seen to extend the survival time of progenitor cells [106]. Other agents including indomethacin and erythropoietin have been used to initiate neurogenesis and angiogenesis [107, 108]. Chemokines such as monocyte chemoattractant protein-1 (MCP-1) can also induce migration of SVZ cells to damaged regions [109]. After an ICH ictus, there is an upregulation of trophic factors like VEGF, hypoxia-inducible factor-1*α*, Ang-1, and Ang-2. These factors will therefore promote some degree of angiogenesis in the brain. Another important compound that induces angiogenesis in hemorrhagic brain is thrombin. Thrombin is studied to induce angiogenesis by activating these trophic factors [110]. Enhancing endogenous neurogenesis and angiogenesis promises to be a novel therapy to improve functional recovery and reduce brain damage. Further studies should therefore be conducted in that respect.

*Cell Transplantation*. Stem cells transplantation has been studied in the treatment of many diseases. Currently, cells being used for preclinical studies include neural stem cell (NSC) or progenitor cells [111], immortalized cells [112], and human mesenchymal stem cells (hMSCs) and human bone marrow stromal cells (HBMSCs) [113]. Neural stem cells therapeutic effect has been clarified by many researchers. These studies focused on how the application of neural stem cells could improve functional recovery and the mechanism of action. One such experiment considered the ability of NSC to produce superoxide dismutase (SOD1) to override ROS stress. Similarly, intravenously transplanted NSC was seen to improve neurologic function [114]. These transplanted cells are able to differentiate into either neurons or glial cell to replace damaged cells [115]. Another preclinical studies on NSC identified the ability of transplanted NSC to reduce neuroinflammation via the downregulation of gamma delta T cells and inflammatory markers but increases in anti-inflammatory markers and regulatory T (Treg) [116]. Mesenchymal stem cells (MSC) have also been intensely studied. The effect of MSC on various stages of ICH has thus been illustrated. For instance, the effect of MSC on BBB protection of ICH was seen to be enormous, in that MSC prevents BBB disruption via upregulation of TNF-*α* stimulated gene/protein 6 (TSG-6). MSC also strengthens the effect of zona occludens-1 and claudin-5 which are integral parts of BBB structure. Another experiment treating ICH with a combination of human mesenchymal stem cell and minimally invasive evacuation recorded improvement of functional recovery [117]. Muse cells are nontumor pluripotent stem cells that have been experimented to be potent in mouse ICH model. Experimental results with implanted muse in mouse model indicated functional recovery models as well as positive test for NeuN and MMP-2. The muse cells were found to be firmly implanted in the mouse brain and differentiated well into neural cells to help functional recovery [118]. Another promising field that has gained attention in recent times is the implantation of induced neurons which are from the cellular reprogramming of fibroblast cells into induced pluripotent stem cells [119]. To get a more efficient protocol for generating neurons from fibroblast that can survive implantation, Pereira et al. delayed that activation of transgene after viral transduction and further treated them with SMAD signaling downregulation and WNT signaling activation molecules. This protocol led to enhancement of neural survival [120]. Similarly, when noggin and SB431542, two inhibitors of SMAD signaling, were inhibited, there was report of conversion of human embryonic cells and iPS [121]. Mesenchymal stem cell from umbilical origin has been found to reduce edema formation and further brain damage when implanted in the 4-day postnatal model. This was done to investigate if mesenchymal stem cell transplantation will offer a treatment option for the very challenging treatment of IVH in premature infants [122]. Cell transplantation therefore presents a promising field which should be explored.

## 3. Translational Therapeutic Drugs

### 3.1. Treating Perihematomal Edema

Brain edema is a frequent occurrence after ICH. The effect of edema cannot be underestimated since it leads to mass effect which further aggravates brain damage which results in death [123]. The process of edema formation has been explained by many different mechanisms with some postulating edema to be caused by imbalance in oncotic pressure or result from BBB disruption by inflammation. The process of edema formation can be categorized into 3 phases. Edema formation in early hours of ICH is attributed to hydrostatic pressure, clot dissolution, and displacement of hematoma serum into the surrounding [124]. The second phase results from inflammatory cascade and thrombin formation, whereas the third phase arises from hemoglobin toxicity due to red blood cell breakdown [123]. Understanding of these mechanisms has led to the development of some potential therapeutic drugs with some currently being used in the clinic as seen in Table 3.

#### 3.1.1. Osmotherapy

*Mannitol*. Mannitol is a commonly used agent in the clinic for the treatment of edema. The efficacy in treating post ICH edema is still controversial. An experiment with five patients highlighted benefit of mannitol in improving clinical outcome [16]. A clinical trial with 2839 acute ICH patients, however, identified mannitol to have minimal significance in ICH recovery [125]. Other additional trials should therefore be done to establish the accurate effect of mannitol on ICH.

*Hypertonic Saline*. Hypertonic solution is another well-known agent for osmotherapy. In a canine ICH model, hypertonic saline was seen to reduce intraparenchymal pressure difference that occurs during ICH with effect lasting for about 3 hours [126] which inevitably leads to the control of edema [126]. Hypertonic saline (23.4%) is seen to control ICP and subsequent herniation of the brain [127, 128]. A retrospective study of the effect of three percent hypertonic saline on ICP and recovery showed the use of hypertonic saline as a feasible treatment of edema and ICP after severe ICH [129].

#### 3.1.2. Neuroinflammation and Oxidative Stress Drugs

After an ICH episode, inflammatory cascade is triggered. This begins with the initial damage of tissues and then the activation of neuroinflammatory factors. Inflammatory factors in turn lead to the disruption of blood-brain barrier. After the integrity of BBB is compromised, circulatory inflammatory factors are able to cross the brain causing further tissue damage which in turn also activates other factors that initiate tissue repair and subsequently lead to recovery [130]. Many studies have therefore targeted the alleviation of inflammation as a novel treatment for ICH [131].

*Celecoxib*. Celecoxib is a selective cyclooxygenase-2 (COX-2) inhibitor. In some animal experiments its mechanism of action has been seen to be effective in anti-inflammation, antioxidation, and neuroprotection. An experiment by Chu et al. suggested that the inhibition of prostaglandin E was probably the reason behind celecoxib's therapeutic property [132]. Smaller randomized control trials (RCT) have been done with patients treated with celecoxib. The result of such study which treated patients with 400 mg/kg of celecoxib for about 7 days showed reduction in hematoma and edema volume, further stressing the safety and efficacy of this drug [133]. Another small trial also stressed on the edema attenuating effect of celecoxib administration. Celecoxib should therefore warrant further larger studies into its application for use in ICH treatment [134].

*Fingolimod*. Fingolimod (FTY720) is a sphingosine-1-phosphate receptor (S1PR) modulator which has received many studies into its role in ICH treatment. The outcomes of these studies have been debatable between those indicating positive outcome and those showing no benefit after acute ICH [135]. Lu et al.'s experiment on the neuroprotective effect of fingolimod is one of such studies which concluded that brain atrophy and neuroinflammation are significantly reduced following fingolimod administration resulting in improvement of neurological function [136]. Another experiment demonstrated that fingolimod significantly lowered lymphocyte count in experimental animals as well as intercellular adhesion molecule-1 (ICAM-1), interferon-*γ* (INF-*γ*), and interleukin-17 (IL-17) count in experimental model [137]. Similarly, a 2-arm clinical trial study of oral fingolimod's effect on perihematomal edema was conducted by Fu et al. In their study, fingolimod was seen to alleviate edema formation and inflammation and improve neurologic outcome [18]. Although fingolimod has protective properties there are also some harmful effects associated with its usage. One way of improving its usage is the use of RP101075 an S1PR modulator agonist with less cardiotoxicity [138]. Fingolimod therefore needs further studies to potentiate its efficacy and reduce its adverse effect in humans.

*NXY-059 (Disufenton Sodium)*. Disufenton sodium is a nitrone with the ability to spin trap free radicals [139]. Due to their carbon-nitrogen bonds, they are able to bind to reactive radical, stabilize them, and prevent them from destroying cell [140]. Many studies with animal model have identified its beneficial effect. For example, studies have identified the therapeutic window for transient and permanent stroke was 2 hrs and 240 mins, respectively, after incidence [141]. Another study indicated that when NXY-059 was used immediately after an embolic ischemia, hemorrhage was likely to occur due to its effect on cerebral vasculature. NXY-059 in combination with tPA however reduced tPA-induced hemorrhage [142]. Similarly, NXY-059G has been seen to have neuroprotective properties and therefore leads to functional recovery when administered [143]. The treatment benefit of NXY-059 was also studied in ICH models. Although rat models showed increase in neurological functions with lower neutrophil infiltration in perihematoma regions, there was no difference between hematoma size for models and control [144]. NXY-059 has undergone different phases of clinical trials. The CHANT phase I trial established NXY-059 as a safe and well tolerated drug for ICH especially at the acute phase within 6 hours [19]. Although well tolerated a trial by Strid et al. indicated that for patients with renal impairment, dosages should be adjusted since nonrenal clearance of NXY-059 is insignificant [145]. Further research into NXY-059 will therefore be helpful in treatment of ICH.

#### 3.1.3. Iron Chelator

*Deferoxamine Mesylate*. Deferoxamine mesylate is an iron chelator approved for detoxication during acute or chronic iron overload [146]. Deferoxamine reduces hemoglobin induced edema, regresses brain atrophy, and improves neurological deficit in animal model [147]. Deferoxamine also reduces the rate of hematoma clearance and affects endogenous ICH response [148]. The dissolution of a hematoma results in the creation of a cavity at the hematoma region. Deferoxamine in a study was found to reduce the size of cavity created after clot resolution. Also, the effect on cells with ferritin and HO-1 present was significantly reduced [149]. Deferoxamine has gone through the phase I trial to determine the maximum tolerated dosage. A dosage of 7 to 62 mg/kg/day was given which was well tolerated with some of the candidates experiencing some adverse effect which is not related to drug [150]. Clinical trials (NCT02175225 and NCT02367248) are currently ongoing to establish the effect of deferoxamine on perihematomal edema.

### 3.2. Treating Hematoma Growth

Rebleeding after an ICH is a common phenomenon which is also a common predictor of outcome after ICH [26]. It is estimated that about 30% of patients will experience rebleeding during early hours of being hospitalized [26]. The treatment of hematoma regrowth could therefore be a surrogate target for ICH treatment (Table 3).

#### 3.2.1. Homeostasis

Recombinant factor VIIa (rFVIIa) has been used for the treatment of blood related disorders like hemophilia and congenital factor VII deficiency [151]. Many experiments have been done to study the effect of this factor on ICH. This is to say, will the administration of Recombinant factor VIIa (rFVIIa) have any effect on hematoma size and if so what effect? In a 90-day RCT study of the effect of Recombinant factor VIIa (rFVIIa) on hematoma size, rFVIIa was seen to reduce the size of hematoma in experimental group as compared to the placebo group [17]. The therapeutic effect of rFVIIa also seems to be dose dependent, with higher doses recording higher efficacy and resulting in some degree of functional recovery in the same group [152].

#### 3.2.2. Anti-Platelet Treatment Reversal

In clinical setting, most patients with ICH have been confirmed to be on at least one antiplatelet medication prior to admission. Antiplatelet has therefore been implicated as a contributing factor of ICH. In an animal model to study the effect of antiplatelet on ICH, it was discovered that there was no significant difference between the pretreated and control group [153]. This is not the case in humans since studies have indicated risk of ICH increases with antiplatelet usage. For example, clopidogrel or ticlopidine usage is seen to have higher risk of ICH than use of aspirin [154]. The reversal of antiplatelet treatment has therefore been controversial although there are some data supporting good outcome after reversal [154, 155]. The third phase of a clinical trial about the effect of platelet administration to patient on antiplatelet therapy indicated that platelet administration was of no much benefit to these patients [156]. Currently, the Neurocritical Care Society treatment guideline recommends desmopressin (0.4 mg/kg IV) on admission and platelet administration for preoperative preparation [157].

#### 3.2.3. Blood Pressure Control

Hypertension with a systolic pressure of BP ⩾ 140/90 mmHg is seen to be the major cause of ICH in about 70% of all cases and also an indication of poor prognosis [158]. The effect of hypertension on post-ICH outcome has therefore been studied in many models. For instance, Sang et al. noted in their experiment that although BP did not lead to spontaneous stroke during their period of study, there was some level of degeneration observed in ICH models despite higher neural stem cell (NSC) recruitment [159]. The impact of lowering blood pressure on hematoma growth has been studied in various trials. For instance, the INTERACT trials identified intensively lowering BP to about 140 mmHg resulting in decrease in chances of rebleeding [21]. Another trial, ADAPT trials (NCT00963976), also focused on acutely lowering BP and its effect on hemodynamic blood flow to the brain and also on hematoma growth [22]. ICH induces a transient disturbance in sympathetic system. As such some therapeutic targets have sort to find the effect of antiadrenergic drugs in the treatment of post-ICH. In CHANT's trial of 303 patients, antihypertensive drugs were associated with decreased edema after BP and hematoma size control [160]. Similarly, other studies have further highlighted the impact of antiadrenergic on the ICH. Another experiment indicated that *β*-blockers, that is, atenolol, slightly improve neurologic outcome after ICH and also prevent complications like pneumonia or SIRS after ICH [161]. Other conflicting trials however indicated that there was no much difference in outcome between various antihypertensive drugs [162]. Another example is the ATTACH trial which found no significant evidence to support effect of lowering BP on hematoma growth and perihematoma edema but encouraged other trials into the effect of aggressive BP lowering on ICH [23].

#### 3.2.4. Hematoma Resolution

*Peroxisome Proliferator-Activated Receptor Gamma (PPARγ)*. PPAR*γ* and its agonist have been instrumental in the treatment of metabolism disorders of glucose and lipid [163, 164]. However, PPAR*γ* has more recently been implicated in the attenuation of inflammatory, oxidative, and excessive phagocytic processes giving indications of its possible use in stroke and ICH treatment. A research by Zhao et al. hypothesized that PPAR*γ* could improve clot resolution by upregulating the phagocyte activity of microglia through CD36 regulation. Activation of the microglia cells will lead to faster uptake of red blood cells and quicker resolution of hematoma [165]. The role of PPAR*γ* in anti-inflammation and antioxidation has also been studied. PPAR*γ* has been found to increase the expression of anti-inflammatory cytokines TGF-*β* and IL-10 and also antioxidative enzymes catalase and superoxide dismutase [166, 167]. Preclinical studies have proven very positive with results indicating PPAR*γ*'s ability to improve clot resolution and provide neuroprotection while improving neurological function [168]. Clinical trials with PPAR*γ* agonist Pioglitazone were started on the safety of Pioglitazone in hematoma and edema resolution [20]. The second phase of the same experiment indicated safety of PPAR*γ* in humans for hematoma resolution (NCT00827892) [20].

### 3.3. Treating Neural Death

The use of stem cell for treatment of various diseases has received major boost in research and clinical trials (Table 3). Stem cells have the ability to differentiate into multiple cells hence their exploration for the treatment of disease like ICH. Treatment focuses either on enhancing longevity and production of endogenous stem cells from the subventricular zone and dentate gyrus or on exogenous transplantation of cells from other sources preferably neural or bone marrow origin [169, 170]. Although cell therapy has demonstrated some treatment benefit in animal model, its application in the clinical setting is still incomplete. Suarez-Monteagudo et al. studied the safety of implanting autologous bone marrow stem cell (BMSC) into perihematomal sequel after 12 months of ICH and stroke. They concluded that BMSC could be well tolerated by patients with no complication [171]. Another study revealed that when autologous bone marrow mononuclear cells were implanted through drainage tube, some degree of neurological function recovery was attained [172]. Similarly, the safety of autologous mesenchymal stem cells (MSC) was tested by Bhasin et al., who concluded that MSC was safe for implantation [173]. Intrathecal administration of bone marrow mononuclear cells (BMMNC) was seen to improve ambulatory function especially for young patient and patient with lesser duration of stroke incidence. This trial (NCT02065778) done on 24 patients after BMMNC was implanted intrathecally and followed up after 4 days for adverse effect and 6 months to 4.5 years for functional recovery [24]. Zhu et al. also performed another trial combining surgery with injection of BMSC, that is, through drainage tube and follow-up with intrathecal injection. A follow-up of treatment showed safety in treatment and decrease in National Institute Stroke Scale (NIHSS) and Rankin scale but increase in Barthel index [174]. There are currently 3 ongoing trials ((NCT02245698/India), (NCT01832428/India), and (NCT01714167/China)) on stem cell therapy which will present a major milestone in the treatment of brain injury through ICH or ischemia.

## 4. Improving Functional Recovery

### 4.1. Prosthetic and Robotic Therapy

One devastating effect of ICH is the impairment of neurological or motor function. The use of prosthesis to enhance movement of the paralytic side has also received some attention. The idea is for the prosthetic part to give support and offer some amount of coordination. Recently, many studies have been done to compare the use of prosthetic training against traditional treadmill gait training. One of such studies revealed significant difference between kinetic abilities at the paretic side for prosthetic part as compared to the treadmill training. It revealed that prosthetic body part combined with treadmill training resulted in early recovery [175]. In another study, the effect of neuroprosthesis on a cerebral palsy child was conducted. The researchers identified some degree of improvement in motor functions as the prosthetic body parts were worn over time suggesting the essence of time in recovery [176]. In other clinical setting, the use of robotic therapy together with conventional therapy showed improvement in motor functions. Robotic parts may serve as a support for plegic side of patients [177]. Similarly, a research by Mehrholz et al. indicated that when paretic patients were given electromechanical and robot-assisted arm, motor function of the hand improved. This was because the robotic part takes up a surrogative role for the plegic hand. They however also stated that care should be taken when interpreting results due to discrepancies in duration intensity in the training for different test subject [178]. Robot-assisted training will therefore serve as a novel treatment for improving motor impairment and improving quality of life [179].

### 4.2. Brain-Computer Interface or Neuroprosthesis

Over the years scientists are trying to find ways to convert electrophysiological waves of the brain into messages that will be used to communicate with the environment [180]. This has led to the development of brain-computer interface (BCI). BCI uses five basic brain impulses, namely, visual evoked potentials, slow cortical potentials, cortical neuronal activity, beta and mu rhythms, and event-related potentials [181]. In patient with severe paralysis (locked in) there is some difficulty in communicating with the environment, although there is some amount of brain activity. The conversion of brains EEG into message which could be understood is therefore a priority of BCI. Different BCI's differ in signal collection, translation of signal, and relay of the information to the user. This disparity makes comparison between different laboratories somewhat difficult if not impossible. As a result, Kübler and Neumann reviewed BCI2000 an online BCI which allows for comparison of different BCI from different laboratory. Their interface differed from other BCI in that it processes neural information online which provide a less costly and effortless access to information data as compared to individualizing BCI [182]. Another study indicated that a locked in syndrome patient due to brain stem stroke can benefit from noninvasive visual P300 speller to enhance communication. This could therefore prove to be of equal benefit to patients who suffer severe paralyses from ICH [183]. There is therefore the need to study more this modality to enable patient with severe paralyses from ICH a chance to communicate with the environment.

### 4.3. Electro-Acupuncture

Traditional Chinese medicine has been practiced for over thousands of years in the treatment of many diseases of which stroke is part [184]. Meta-analytical studies revealed different approaches employed for stroke treatment, with varying results from improvement, adverse reactions to treatment, and sometimes even death [185]. In recent years, scientists have tried to blend the use of TCM with other scientific methods. For instance, Zhao and Yu explored the effect of cranial acupuncture on serum IL-6 content. They reported an improvement in nervous function and decrease in serum IL-6 [186]. Electroacupuncture (EA) is the combination of acupuncture and electrical stimulation. EA has been identified to attenuate the disruption of BBB after stroke. In a rat model experiment to examine the effect of EA on BBB, Wu et al. used Evans blue dye as a marker of extent of BBB disruption. They concluded that EA has BBB protective ability [187]. EA at acupoints “GV26” and “GV20” for 30 min has been found to have an antioxidant property in rat model. Results from Zhong et al. experiment showed improvement in mitochondrial function accompanied with succinic dehydrogenase, NADH dehydrogenase, and cytochrome C oxidase activity increase. Results therefore indicated an elevation respiratory enzymes activity and a decrease in reactive oxygen species (ROS) and production [188]. The influence of EA on growth factors has also been studied. EA has been seen to increase cerebral blood flow [189]. Although some amounts of clinical studies have been done on EA (Table 3), the evaluations of results have been moderate [190]. The need to carry out further accurate trials and data collection is needed in ensuring the practicality of EA in ICH treatment.

## 5. Limitations of Translating Preclinical Studies

**Figure 2:**
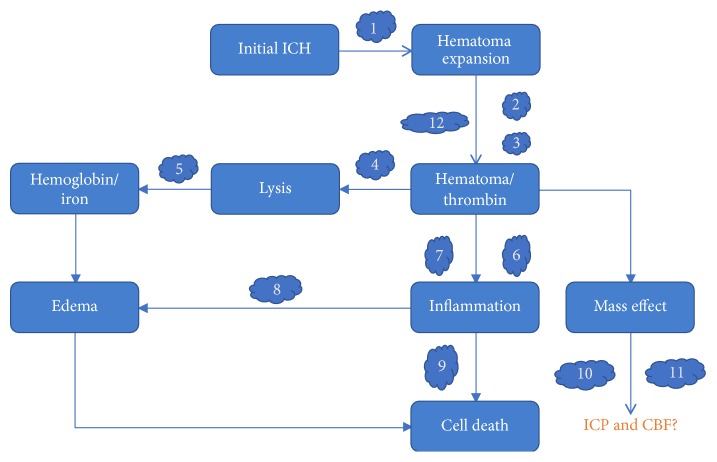
Potential therapeutic targets and treatments for ICH: 1. beta blocker; 2. rFVII; 3. desmoplasmin; 4. PPAR*γ*; 5. deferoxamine; 6. celecoxib; 7. NXY-059; 8. fingolimod; 9. stem cell therapy; 10. mannitol; 11. hypertonic saline; 12. Pioglitazone.

Preclinical animal model studies have enabled us to understand ICH disease process to some extent leading to some amount of translational application. With our current understanding of ICH, conservative treatment could be clinically targeted at different stages of the condition as shown in Figure 2. However, there is more to the treatment of ICH than the scope of this review. There are still some limitations that are yet to be overcome to propel these preclinical studies into clinical usage. Till date, the creation of an ideal model that fully mimics the entire disease process of ICH in humans is still a challenge. Most of the current models fail to develop a model that fully incorporates epidemiological and nonepidemiological factors of ICH. Another challenging factor is minimizing human or experimental errors. Although most of the preclinical drugs have experienced some level of success during experimentations in ICH models, these drugs are yet to be experimented in large animals or humans. Although some of these experimental drugs have already been used in clinical trials in the treatment of other diseases their advancement into ICH related clinical trial is yet to receive a breakthrough. This is due lack of drug specificity and other deleterious side effects associated with those trials. For instance, clinical trials of curcumin have revealed DNA fragmentation in the presence of P450 and other deleterious drug interactions. It is therefore prudent to fully understand the mechanism of action of experimental drugs before trying them on humans [191, 192]. Furthermore, although some pathways have been studied as therapeutic targets, the actual mechanisms underlying these pathways are yet to be understood. For example, inhibition of TLR4 signaling has been identified to be a preventive or treatment mechanism for ICH. Inhibition of TLR4 has been found to be either by deletion of TLR4 gene or by anti-TLR4 antibodies; however, there is still the need for studies into the specific antagonist for TLR4. Knowledge about the most critical ligand as well as the specificity of TLR4 signaling across similar cells is yet to be understood [193]. It is therefore not surprising that although a milestone in animal studies has been achieved, we are yet to see translation into clinical trials. Experimental errors and inconsistencies in result across different laboratories also make it a challenge to translate studies of experimental drugs into clinical use. This might be due to lack of transparency and accurate data collection of experimental data. These inconsistencies will therefore mean that although one experimental result showed positive results, other laboratories might fail to replicate these results, making clinical applications impossible. We therefore are of the view that extra studies are needed in small and large animal to unravel the mysteries behind ICH disease process as well as improve limitations of preclinical studies into clinical applications.

## 6. Conclusion

In this review, we looked at some conservative treatment options of ICH from preclinical studies as seen in Figure 1. We also focused on some translational studies and trials that are ongoing and those completed. Although some novel treatment therapies have been developed to treat ICH, there still remains a lot to be discovered. There are still new drugs that have been experimented to be efficient in small animal models but are yet to be tried in large animals (Table 2) and then the clinics. There is the need for in-depth studies into these new drugs. Furthermore, limitations associated with translational studies of these potential therapeutic modalities should be curtailed, to enrich the treatment options of this complicated condition.

## Figures and Tables

**Table 1:** Common models for studying ICH.

ICH Models	Merits	Demerits
Autologous blood injection	Easy to perform	
	Easy to reproduce	Needle trail reflux
	Hematoma size fixed	Cannot mimic rebleeding
	Mimics lobar hemorrhage	Short edema peak time

Collagenase injection	Mimics rebleeding and hematoma expansion	Possible cellular toxicity
	No reflux needle trail Simulating perforating artery rupture	Inconsistent hemorrhage
		Excessive neural damage

Microballoon inflation	For studying mass effect	Minimal damage observed
	Injury confined to inflation site	Only for simulating mass effect

**Table 2:** Preclinical drugs and their potential function.

Experimental drugs	Target	Function
Curcumin	Inhibit MMP-9, suppress TNF-*α* and iNOS	Neuroprotection, edema alleviation
Progesterone	Inhibit MMP-9, restore (BDNF)mRNA, NaK-ATPase mRNA, MAP, and ChAT	Neuroprotection, decrease glial cell and edema formation
(−)-Epicatechin	Upregulate Nrf2	Alleviates oxidative stress,
Prostaglandin E2	Downregulate HMGB1, interleukin-1*β* and Src kinase expression	Prevent edema formation, neuroprotection
Melatonin	Downregulate MMP-2 and MMP-9	Neuroprotection and edema alleviation
Imatinib	Downregulate PDGFR-*β*	Neuroprotection
Sparstolonin	Inhibit TLR2/TLR4 and NF‐*κ*B	Neuroprotection
Dexamethasone	Increase Bcl-2/Bax, downregulate ICAM-1 and MMP-9	Edema alleviation
Aprotinin	Inhibit plasma kallikrein	Prevents rebleeding and edema formation

**Table 3:** Previous and current clinical trials.

Agent	Name of study	Result	Trial Number	Target
Mannitol		No significance		Edema [16]

rFVIIa	FAST	Ongoing	NCT00127283	Hematoma regrowth [17]

Fingolimod		Phase 2	NCT02002390	Edema [18]

NXYO59	CHANT	No significance		Free radical [19]

Deferoxamine	DFO-ICH	Ongoing	NCT02175225	Iron chelation & perihematomal edema
			NCT02367248	

Pioglitazone	SHRINC	Phase 3	NCT00827892	PPAR*γ*-agonist [20]

Celecoxib	ACE-ICH	Ongoing		cyclooxygenase-2

Hypertension	CHANT	Ongoing		Hematoma regrowth & BP control [20]
	INTERACT	Completed		Hematoma regrowth & BP control [21]
	ADAPT	Completed		Hematoma regrowth & BP control [22]
	ATTACH	No significance		Hematoma regrowth & BP control [23]

Stem cell		Ongoing	NCT02065778 NCT02245698 NCT01832428 NCT01714167	Many targets [24]

Electroacupuncture		Ongoing		Many targets
